# Analysis of COVID-19 Name Varieties in Chinese Mass and Social Media

**DOI:** 10.3390/ijerph18189850

**Published:** 2021-09-18

**Authors:** Hongjie Dong, Minli Zhou, Dewei Che, Huiying Zhang, Adams Bodomo

**Affiliations:** 1 School of Liberal Arts, Xi’an University, Xi’an 710065, China; donghongjie@xawl.edu.cn; 2 Department of Liberal Arts, Guangdong University of Education, Guangzhou 510303, China; minlizhou@163.com; 3 Faculty of Philological and Cultural Studies, University of Vienna, 1090 Vienna, Austria; adams.bodomo@univie.ac.at; 4 School of Liberal Arts, Shaanxi Normal University, Xi’an 710062, China; zhanghuiying@snnu.edu.cn

**Keywords:** COVID-19, severe viral pneumonia, new coronavirus epidemic, disease name varieties, distribution across media, usage timelines, daily frequencies, word forms

## Abstract

The sudden appearance of a new epidemic disease in China created the need for names identifying that disease. Between December 2019 and January 2020, a variety of severe pneumonia-related disease names suddenly appeared, and more name varieties kept coming up afterwards. To better understand the introduction and spread of these names, 16 different COVID-19-related name varieties were selected covering the period from the end of December 2019, when the epidemic started, to mid-March 2020, a moment at which the term competition had stabilized. By way of big data analysis, the initiation and distribution of the 16 names across the media landscape was traced with regard to the impact of different media platforms, while the distribution frequency of each of the selected terms was mapped, resulting in a distinction of three groups of disease names, each with a different media and time profile. The results were discussed based on the hypotheses of disease confusion by name variety and management failures in absence of clear language governance at the national and global levels. The analysis of the data led to a refutation of both hypotheses. Based on this discussion, the study offers empirically based suggestions for the WHO in their naming practices and further research.

## 1. Introduction

At the end of 2019, a new severe respiratory disease swept through China. This disease was caused by a new and “unknown” virus, which on 7 January 2020 was recognized as a “new coronavirus”, accepted as “contagious” on 20 January, and officially referred to as COVID-19 by the WHO on 11 February. Initially, all levels of public communication, including governmental, commercial, and social media, adopted a variety of terms to refer to this new disease [1]. Some of the names were very popular whereas others attracted marginal attention. Some terms were used at high daily frequencies whereas others seemed to disappear after a certain period of time. Against this background, we address four questions in this paper, including the number and form of names used to refer to this new viral disease, the extent of daily frequency variation, their distribution across media type, and the build-up of their timelines. We employed internet search technology tracing individual disease names across a wide variety of media sources. These online data include a selection of 16 names that appeared online somewhere before the announcement of the official WHO name. No new names were added after that date.

In Section 2 we provide an overview of discussions related to disease name selection in China, prominent among these being perceived problems of confusion, when different names for the same disease are in use, and proposals for betterment of governance and disease name planning in China. In Section 3, we detail the research methods and in Section 4 we present the results. We first report on the spread of the selected terms across media types (Section 4.1), detail the timelines of each term (Section 4.2), and analyze the relation between frequency of use and length of word form (Section 4.3). In Section 5, which is the discussion section, we analyze the various data in relation to time of name introduction and the competition among name varieties. We conclude by offering empirically based suggestions for WHO naming practices and further research.

## 2. Naming Viruses and Diseases

It was on 30 January 2020 that the World Health Organization (WHO) expressed international concern about the spread of the virus worldwide since its inception in December 2019, which means that until that time the disease was treated as an epidemic that had not yet attained pandemic proportions. The significant points in development were the identification of the virus that caused the disease as a “new coronavirus” on 7 January, the sharing of the genetic sequencing by China with the WHO on 11 January, and recognition of human-to-human transition on 20 January. The organization did not declare the spread of the virus a pandemic until 11 March 2020. (https://www.who.int/emergencies/diseases/novel-coronavirus-2019, accessed on 15 April 2021).

In the beginning of February 2020, the disease was still unknown and was spreading without having received an official name, while a variety of names was used to refer to the new disease. It was only on 11 February 2020 that the name was defined by the WHO as COVID-19. In the following analysis, we provide an overview of international and Chinese institutions and persons officially involved in disease naming. We also address the issue of naming on social media.

The International Committee on Taxonomy of Viruses (ICTV) works on the classification of all known viruses. It is from the environment of the ICTV that the name Coronavirus originates, one of a variety of viruses classified as RNA viruses, which cause respiratory problems in birds and humans [2]. The term “coronavirus”, *guānzhuàng bìngdú* in Chinese, follows this taxonomic tradition and is a term that is used widely in nontechnical publications such as newspapers, news reports, and online media. It is also the ICTV that advocates a name for the new virus that is neutral and can be used by the WHO. The ICTV, WHO, and other international organizations intend to avoid names that are associated with a person, a location, or a particular industry [3]. As known already, it is through the WHO that the name COVID-19 was introduced. Real life data presented in this study show the conflict that exists between practical naming in actual circumstances and nonpartisan considerations by international and Chinese institutions [4,5]. The influence of the international institutions can be seen in the choice of the English name, COVID-19, by the Chinese government and the mass media [6].

In a 1997 paper, a group of Chinese authors discussed how to improve the standardization and accuracy of disease terminology [7]. Based on the data from a military hospital, Liu Yaxian and his colleagues outlined nine naming issues in medical practice in the Chinese language. Having examined potentially confusing abbreviations and multiple names for one disease, they suggested introducing language policies in Chinese hospitals, including regular ICD (International Classification of Diseases) training and the implementation of more strict medical term standardization.

Another author, Ran Wang [8], noticed the lack of a naming system for infectious diseases in China and identified complications arising for their use in emergencies. He used the case of the 2009 Influenza A (H1N1) pandemic as an example, which started in the United States and spread worldwide killing more than half a million people. In Chinese, this virus was called Pig Flu, generally considered an improper disease name, which, as a result of nonguided naming, caused great damage to the pork industry. He further examined the existing language policy toward infectious disease naming, stressed the need for information transparency, and proposed explicit naming procedures, including naming agents, supervisors, and sanctions.

This issue was further investigated by others, who discussed details of the classification mechanism of disease naming [9]. Wang Yu and Jin Zhaoxia examined the naming practices and deviations from ICD (International Classification of Diseases) terminology based on their clinical data in a Xinjiang Hospital. They argued that there is a difference between disease classification and disease naming. Scientific terminology is useful for researchers but not necessarily practical in everyday situations. They suggested naming a disease within an existing classification configuration. By doing so, the risks posed by improper medical names would be reduced and serious damage could be avoided.

Against the background of the spread of the new virus, warnings were also published regarding problems which could occur by insensitive naming [10]. The example given was the name Middle East Respiratory Syndrome Coronavirus (MERS-COV). It was argued that the name is stigmatizing for the geographical area concerned. Tweets emerged calling it an “insult” and an “ethnic slur” after the journal Science revealed this name in an online story. The WHO had to adopt a more neutral name and came up with “novel Coronavirus”, abbreviated either as NCoV or nCoV. The impact of infectious disease naming during emergencies was also brought up by Wang Chunhui, who argued that a variety of different names causes confusion [11]. At the beginning of the COVID-19 pandemic, he noticed, the disease was referred to as *fèiyán*, “pneumonia”, and also as *bìngdú*, “virus”, whereas in English terms such as SARS-CoV-2, COVID 19-nCoV, and COVID-19 were used, which in Chinese were rendered as *Xīnguān fèiyán*, “New coronavirus pneumonia”, among many others, as we will see shortly.

Qi Shen and Minghao Kang, finally, emphasized requirements for language governance during major public health emergencies [12]. They observed that global public health emergencies, such as COVID-19, pose a serious challenge to national level language competence in China. China needs to be able to participate in global governance, understood as the way in which international formal institutions interact with civil society. It is therefore imperative for China, they argued, to undertake language governance planning. Inspired by the theories of language governance and language planning, they proposed a framework of governance typologies leading to governance practices and language planning for public health emergencies. They argued that, in cases like this, language policy should consider a series of steps varying from prevention and emergency preparation to ways of disease monitoring, public warnings, emergency handling, and rescue operations.

We conclude this overview with four recent articles addressing issues relevant to our study. Two of these present concepts that are relevant but cannot be fully addressed in our study [13,14]. The first of these is a literature overview on the role of “word of mouth” in electronic settings (eWOM). The study tackled the tourism industry but some of the findings can be related to our work. The second conceptual paper studied the effect of sociological variables on internet users’ opinions and focused on the use of ad-blockers by different generations. We revisit these issues in the discussion section, such as the strength of existing social ties as well as the importance of action-based information as opposed to opinion-based news items.

Another recent empirical study, Zhu et al. [15], measured the attention of Chinese netizens to COVID-19 via a self-built corpus of Weibo users, in which they searched for COVID-19-related keywords. The study selected the period from 1 January to 12 February 2020 for data collection and listed 42 COVID-19-related items, of which the most frequent three, “epidemic situation”, “mask”, and “pneumonia”, represented 79.2% of their data and a term such as *Shìwèi*, the Chinese abbreviation for the WHO, occurred less than 1% and the “Huanan seafood market” less than 0.5%. The timeline for their data shows a lack of attention to the disease until attention was taken-up at the national level and the Wuhan lockdown occurred. Liu et al. [16] used a similar strategy. These authors used the keyword “coronavirus” to extract related news articles from the WiseSearch database and analyzed the content these articles represented. The data were collected between 1 January 2020 and 20 February 2020. The study distinguished 20 topics distributed across nine themes, three of which counted for 60% of the data. They further presented a “time series of news streams” which shows strong similarity to the time series presented by Zhu et al. [14], a lack of news items at the beginning of January and a strong increase in news items starting on 19 January when central authorities stepped in as regulators of “prevention and control”.

Both recent studies used the concept of keywords to attract information from a database and in that sense our data collection is very similar. Our keywords are a selection of disease name varieties for each of which a time series can be plotted. Our data support our general hypothesis that name varieties compete with each other in terms of the timeline along which the various names were introduced, the media type involved, the shape of the timeline of each individual name, as well as the type of word format applied. The question that follows therefore is the extent to which the data collected by this study are similar to the time series published by these two studies.

This overview of “coronavirus”-related issues also suggests three individual hypotheses that our work can address. The first of these, as mentioned, is the claim that timelines of our study are highly similar to those presented in the two recent studies. The second hypothesis is the probability that the presence of different names for the same disease will cause confusion and have a negative effect on communication practices. The third hypothesis addresses the issue of global governance, which states that China is linguistically not prepared to interact with international institutions.

Both international and national forces are involved in the selection of disease names in China. What is not mentioned so far is the potential role of social media in this naming process. In online communities, netizens need names to express their ideas, emotions, and concerns, and they most likely will not and cannot wait until the medical authorities have figured out the complexity of an epidemic situation. We therefore expect creative contributions by participants in social media. Given this variety of international and national influences, the main issue we need to address is the road Chinese disease terminology developed in the first months of the new spreading disease.

## 3. Method

Given the constraints imposed by a large-scale pneumonia outbreak-related national lockdown in China, which started on 23 January 2020, the data for this study were collected online. We used modern online search technology to build a database of names in use for the new coronavirus-related disease as these started to appear in mass and social media. This means that we were looking for Chinese names for the “new viral” severe pneumonia and did not include technical English language names such as SARS-CoV-2, unless these were officially recognized by Chinese authorities and featured in Chinese language media.

The data collection covered a period of eight weeks, from the end of December 2019 to the middle of March 2020. The most prominent names and their varieties were monitored until 11 February, the date on which the WHO proclaimed the official name of the disease as COVID-19. This latter name was the last term incorporated into our corona disease name set; no new names were added after that date, resulting in a basic set of 16 names, which were used as reference to the new pneumonia disease and, during that time, caused such extensive and emotional harm for the Chinese people.

For the data collection, we relied on the Qingbo Big Data site, a popular big data platform for Chinese news media. The site covers various media such as the People’s Daily Online, Sohu News, and China News, as well as online social media such as WeChat and Weibo, not forgetting short video platforms. The platform monitors these sites in real-time and collects information from the entire network. The platform provides a variety of data that can be used to trace and analyze emergencies (such as a new disease), determine the extent of event attention, and obtain trends in event development. Thanks to this modern technology, a total of more than 88 million sentence tokens were collected. The Chinese, Pinyin reference, and English translation of all 14 Chinese names are illustrated in Table 1 below. The two English names are listed there too.

The listing of the time of introduction of the 16 names shows that the 14 Chinese names, with one exception, all shared the disease indication “pneumonia”, *fèiyán* in Chinese (Table 2). The exception is an abbreviation of a longer name and therefore still immediately recognized in context. In the early days, when it was not clear yet what kind of pneumonia one actually encountered, the names that appeared in our data were “new type”, “China”, “Wuhan” (later also “Han” occurred as an abbreviated form of “Wuhan”), “unknown”, and “wild animal”. After 9 January, it became clear that there was a connection with a coronavirus, which was expressed with names containing phrases such as “novel coronavirus”, “novel viral”, “new coronavirus”, “new viral”, “novel coronavirus” and “viral pneumonia” related to “wild animals/game”.

The two English names in the list, “COVID-19” and “NCP”, are the official names published by the WHO on 11 February 2020 (https://www.who.int/dg/speeches/detail/who-director-general-s-remarks-at-the-media-briefing-on-2019-ncov-on-11-february-2020, accessed on 15 April 2021) and “NCP” is the official English name for the disease announced as such by the NHCC (National Health Commission of China) on 7 February 2020 (http://www.gov.cn/zhengce/zhengceku/2020-02/08/content_5476248.htm, accessed on 15 April 2021). The term was attested in a particular subset of our data on 8 February.

During the data processing period, it was necessary to exclude certain high frequency character combinations which were identical or partly identical to the name of the virus and made it difficult to capture the related data. For instance, over 2000 companies in China contain the characters *Xīnguān*, “New Crown”, whose character combination is identical with one of the name varieties, namely, No. 7 *Xīnguān*, “Novel coronavirus” and part of name No. 5 *Xīnguān fèiyán*, “New coronavirus pneumonia”. Despite the need for restrictions in data collection, we built a corpus covering 57 media, comprising 88,824,586 sentence tokens, a number, as will be understood, which is only manageable with modern big data processing tools. These techniques also make it possible for researchers to repeat our study, a situation that exists as long as access to the big data database remains available.

## 4. Results

**Figure 1:**
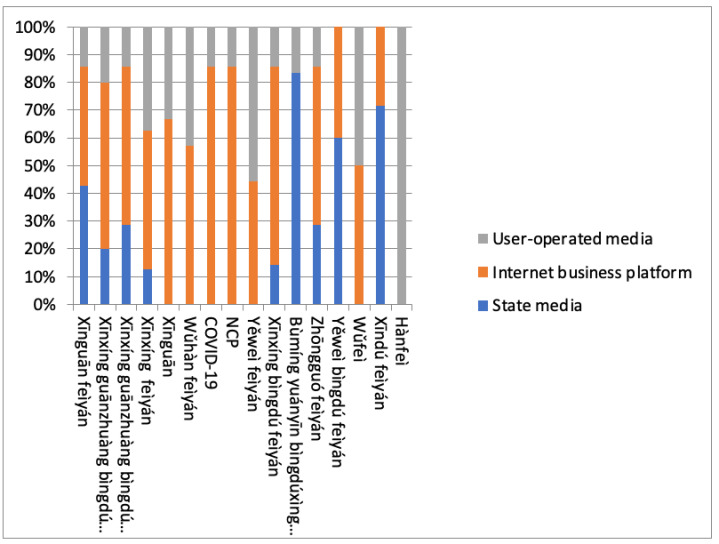
Frequency distribution of names across active channels.

The 16 disease names introduced were spread across 51 active channels. These media channels represent three different stakeholders: state media, commercial internet platforms, and social media. State media are run by the Chinese government, such as “Xinhua Net”, “China Economic Net”, “Taihai Net”, “China News Net”, and others, a total of 17 media. Commercial internet platforms are founded by commercial companies, with the purpose of producing information and gathering user data. Examples are “Sohu News”, “The Paper”, “Zhongshi News”, “Los Angeles Life Interaction”, and others, a total of 16 news sites. Social media provides an opportunity for individual netizens to share their opinions and allows them to interact with other participants in a social network. Weibo is the best-known example of a social channel (comparable to Facebook in the West) in all 18 social media platforms. Figure 1 shows the distribution of the 16 names across the 51 active channels we traced.

The results are discussed in three steps; first we look in more detail at the data presented in Figure 1 in order to clarify the role of each of the three media types played in the distribution story (Section 4.1); we then analyze the timelines of each name to study the nature of their character (Section 4.2); and finally, we look at the relation between frequency of use and word length, assuming that shorter terms are used more frequently than longer ones (Section 4.3).

### 4.1. Terms Shared across Media

Using the extent to which terms are shared across media channels as a criterion, the 16 terms fall into three groups. We look first at names shared by state, commercial, and social media (Section 4.1.1), and then at those shared across commercial and social media with no references in state media (Section 4.1.2). The remaining four names and their distributions are discussed in the third section (Section 4.1.3).

#### 4.1.1. Shared across All Media

The six terms shared across the three media, state, commercial, and social are, in order of frequency:

No. 5 “New coronavirus pneumonia”;

No. 4 “New type pneumonia”;

No. 1 “Novel coronavirus pneumonia”;

No. 2 “Pneumonia caused by novel coronavirus”;

No. 3 “Novel viral pneumonia”; and

No. 14 “China pneumonia”.

The data presented in Table 3 make clear that in terms of frequency, the six terms fall into two groups, one group of three terms with very high frequency of occurrence and a second group of three with much lesser frequencies. The first group of three had two members with high frequencies of close to two million total occurrences and one member with an outstanding position of more than four million. This high number was for name No. 5. *Xīnguān fèiyán*, “New coronavirus pneumonia”. An example of this name appears later in the results section. Here we give an example of another member of the high frequency group, name No. 1 *Xīnxíng guānzhuàng bìngdú fèiyán*, “Novel coronavirus pneumonia”, which was found in the People’s Daily dated 7 February 2020, just before the publication of the official coronavirus name by the WHO:

(1) Yīwèi xīnxíng guānzhuàng bìngdú fèiyán zhìyùzhě de zhēnshí tǐhuì.

“The real experience of a person cured of Novel coronavirus pneumonia.”

(*Rénmín wǎng*; People’s Daily Online, 7 February 2020)

Total weekly frequencies among the members of the second group, in contrast, varied between 300,000 and 3,000. Name No. 2, *Xīnxíng guānzhuàng bìngdú gǎnrǎn de fèiyán*, “Pneumonia caused by novel coronavirus”, was introduced around 19 January by the NHCC and taken up by other official institutions, for instance, the National Health Commission of Wuhan (NHCW). Official names generally were announced in the form of a letter or a press conference, and around 19 January it was known that we were dealing with a “coronavirus”, *guānzhuàng bìngdú* ‘, that was *xīnxíng*, “novel”, causing *fèiyán*, “pneumonia” and was probably *gǎnrǎn*, “contagious”. An example taken from China Youth Net on 20 January 2020 illustrates its use:

(2) Xīnxíng guānzhuàng bìngdú gǎnrǎn de fèiyán yǒu nǎxiē zhèngzhuàng? Chángtú jiědú lái le.

“What are the symptoms of *Pneumonia caused by the novel coronavirus*? Here comes the interpretation of the long picture.”

(*Zhōngguó Qīngnián Wǎng*; China Youth Net, 20 January 2020)

Table 3 further demonstrates that the usage of these six terms was not equally shared across the three media types. Close to equal sharing existed between state and commercial for the most frequent term, No. 5 “New coronavirus pneumonia”, with 42% and 44% for these two media, respectively. As these figures indicate the high-frequency name was less popular on social media with a figure no higher than 14%. For all six terms, the commercial sites represented the widest use. The commercial media percentages were, from high to low, for No. 3 “Novel viral pneumonia” 70%, No. 2 “Pneumonia caused by novel coronavirus” 60%, and an equal 55% for No. 1 “Novel coronavirus pneumonia” and No. 14 “China pneumonia”, and finally, No. 4 “New type pneumonia” with 50%.

No. 14. “China pneumonia”, the least frequently used term of all six, interestingly, occurred more frequently in state media with 28% than in social media with 17%, a difference that needs further scrutiny, which is done in the next section where we present data of frequency development for each of the 16 names across time.

#### 4.1.2. Shared by Commercial and Social Networks

Commercial and social networks, too, shared six terms. For clarity, none of these six terms were found in official state-controlled media. The six terms of this type were in the following order of frequency:

No. 7 “Novel coronavirus”;

No. 8 “Wuhan pneumonia”;

No. 15 COVID-19;

No. 16. NCP;

No. 12 “Wild animal pneumonia”; and

No. 9 “Wu P”.

As shown in Table 4, the total of the average weekly frequencies of these terms ranged from 161,000 to 1000. Name No.9 *Wǔfèi,* the shortened form of No. 8. *Wǔhàn fèiyán*, “Wuhan pneumonia”, had an eight-week total frequency of 124,000. The top daily frequency among the six shared items of this selection was for name No. 7 *Xīnguān*, “Novel coronavirus”, with a total weekly frequency of 161,000. Name No. 12 *Yěwèi fèiyán*, “Wild animal pneumonia”, ended up at the lower end of these data with a total frequency of 22,000. The two English terms, No. 15 COVID-19 and No. 16 NCP, had frequencies of, respectively, 90,000 and 60,000, taking up the middle position among these six terms shared by commercial companies and social media.

Four of these six disease names were more widely used in commercial sources. Outstanding among these were the two English terms, COVID-19 and NCP, with a majority occurrence of 85% in commercial environments. A closer look showed that their uses were mainly supported by Chinese language media predominantly intended for a Taiwanese or North American Chinese audience, such as the popular channel “Los Angeles Life Interaction”. Terms No. 7 “Novel coronavirus” and No. 8 “Wuhan pneumonia”, too, were dominantly used in commercial media with 68% and 58%, respectively, allowing social media one-third to almost half of the traced occurrences. In contrast, No. 12 “Wild animal pneumonia” was used somewhat more extensively in social media with 56%, showing social awareness of the often-quoted wet market origin of the new virus. Equal distribution across both media was the case for the, less frequently used, name No. 9 *Wǔfèi* “Wu P(neumonia)”, reflecting a certain but limited need for very short and easy to use terms in both nonofficial media.

#### 4.1.3. The Remaining Four Disease Names

Of the remaining four disease names, three were mainly used in official media, whereas one little-used term occurred in social media exchanges only. The four names in terms of frequencies were:

No. 13 “Unknown viral pneumonia”;

No. 11 “Viral pneumonia caused by wild animals”;

No. 6 “New viral pneumonia” and;

No. 10 *Hànfèi* “Han P(neumonia)”.

The data listed in Table 5 show that the total numbers varied between three-thousand-two-hundred and nineteen occurrences. The social media data contained one term that was not used by any of the other two media, state or commercial. This term was an easy choice for social media participants, those who can read and write Chinese that is. The term was not used by state-sponsored and commercial sites, probably because this abbreviation shares a high degree of similarity with local hospitals’ names, such as Wuhan Pulmonary Disease Hospital, short for “Hanfei”.

The first of the names in Table 5, No. 13 *Bùmíng yuányīn bìngdúxìng fèiyán*, “Unknown viral pneumonia”, was an official name and not reported by any commercial site. “State” had a strong majority stake of 82%. It was shared to some extent by social media. This is one of the early state-designed disease names, reflecting the discovery of a new, “unknown” yet, coronavirus. The name was only marginally supported by users of social media and neglected by commercial sites. Businesses probably do not like the term “unknown”, which sounds not very business-like. In business environments things tend to have clear sources and straight forward values. Here is an example of its use taken from the Sina.com website dated 6 January 2020:

(3) Wǔhàn wèihé xiàn bùmíng yuányīn bìngdúxìng fèiyán? Shìjíkòng zhōngxīn huíyìng “Wǔhànshì wèijiànwěi guānyú bùmíng yuányīn de bìngdúxìng fèiyán qíngkuàng tōngbào”

“Why is there ‘Unknown viral pneumonia’ in Wuhan? The Municipal Center for Disease Control and Prevention responded to the ‘Notice of Wuhan Municipal Health Commission on Unknown viral pneumonia’”

(*Xīnlàng Wǎng*; Sina.com, 6 January 2020) (The use of the subordinating particle *de* in the second quote of the disease name makes that into a descriptive term more than a disease name.)

The status of term No. 11 *Yěwèi bìngdú fèiyán*, “Viral pneumonia caused by wild animals”, was shared by state-sponsored and commercial media with a majority of 60% for use in official sites. This result is puzzling, since it is not an official term, but its use in official media is related to its appearance in the CCTV News program “CCTV Comment” on 23 January 2020. In that program, it was stated that some netizens by selecting this term wished to warn others about the danger of eating game food and encouraged them “to refuse to eat wild animals”. The program also expressed the view that “the virus should be named more scientifically”. The main point, however, was that it urged people to learn from the *Yěwèi bìngdú fèiyán*, “Viral pneumonia caused by wild animals” case.

This report was reprinted and reposted in large numbers by other mainstream media such as People’s Daily Online, Sina.com, Tencent.com, and others, and was followed and discussed on Weibo. Within four days, the number of Weibo comments on this topic reached 2756. As a result of this, the spread of name No. 11 is different from other nonofficial name varieties, meaning that critical reports by mainstream media are a factor affecting the spread of disease name varieties.

The last term supported by state sponsored media, name No. 6 *Xīndú fèiyán*, “New viral pneumonia”, is a four-character name abbreviated from *Xīnguān*, “new corona”, and *bìngdú*, “virus, combining the first and the last character of these two words to create the abbreviation *Xīndú*, “new viral”. This new term was not taken up by netizens and was only used by commercial sites in 29% of the cases noted, showing the strong preference for this term by state media, which were responsible for the remaining 71% of its total frequency.

The main force behind the acceptance and spread of the 16 new disease names were commercial websites. Chinese governmental sites created or accepted nine of the 19 terms but, with the exception of three, the most frequently used six names (Table 3) were in the majority employed by commercial sites. The three remaining official terms were less frequently used (Table 5). Social media excelled in one term only, name No. 10 *Hànfèi*, “Han P”; it further shared one term with commercial sites, name No. 9 *Wǔfèi*, “Wu P”, and used one other term dominantly, No.12 *Yěwèi fèiyán*, “Wild animal pneumonia”.

### 4.2. Disease Names across Time

For each of the sixteen disease names studied, statistics of daily frequencies and weekly averages are available. The frequencies quoted in the previous section were the total scores of the weekly averages across an eight-week period or across the number of weeks of actual use. In this section, we will use the daily frequencies to compare the timelines of the 16 names we selected. Each timeline characterizes the occurrence and development of the frequency of use of a disease name. For instance, the maximum frequency disease name No. 5 *Xīnguān fèiyán*, “New coronavirus pneumonia”, first appeared on 18 January and soon thereafter on the 23rd reached a peak of 200,000 occurrences, a peak moment which coincided with the official announcement of the Wuhan lockdown.

That announcement occurred at 2 o’clock in the morning of that day signaling the urgency of the situation. The announcement stated that the lockdown would take effect at 10 o’clock that same day, which must have caused great anxiety among both the local and wider population. This is particularly understandable when it is realized that the lockdown and limitation of movement occurred two days before the Chinese New Year celebrations of that year. In that context, it is understandable that together with the encompassing chaos, a flood of urgent and frantic interpersonal communications appeared.

**Figure 2:**
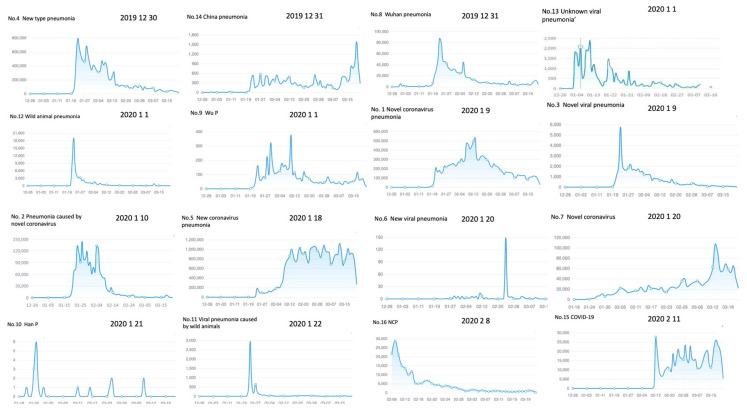
Timelines of 16 coronavirus-related infectious disease names.

The following two weeks saw increasing use of name No. 5 “New coronavirus pneumonia” at levels of several hundred thousand hits a day, at the end of which reaching a level of one million. With ups and downs, name No. 5 “New coronavirus pneumonia” remained on that high a level until the end of the observation period. All media types confirmed its use with state and commercial media contributing most of the data (Table 3; Figure 2).

Name No. 5′s configuration, slow build-up, and remaining high level was quite unique and can only be compared with the much later occurrence of the international official name for the new virus disease, COVID-19, name No. 15 in our data, which appeared after 11 February and remained in use at a somewhat lower but relatively high level of 10,000 to 20,000 daily occurrences. The term was not used by official media in China, since they are required to use the official Chinese names. Commercial and social media do not have such restrictions.

Here is an example of the use of COVID-19 in a Chinese text. The example was taken from the *Wǎngyì xīnwén* news site, which is run by NetEase Inc., a Chinese technology company providing online services centered on content. The company is listed at the Nasdaq stock exchange. The example reads:

(4) Dào jīntiān, quánqiú 180 duō gè guójiā dōu yǐjīng bàogào le COVID-19 bìnglì.

“As of today, more than 180 countries around the world have reported COVID-19 cases.”

(*Wǎngyì xīnwén*, ‘Net-ease News’, 22 March 2020)

In contrast to the two high daily frequency timelines, No. 5 and No. 15, disease name No. 4 *Xīnxíng fèiyán*, “New type pneumonia”, which was the first official name for the disease and first appeared in our data at 30 December 2019, has a quite different timeline pattern. We characterize this pattern as rise-and-fall and found that six other items, five Chinese and one English name, shared this pattern. Together these seven timelines represent 44% of the data.

Name No. 4 “New type pneumonia” reached a peak during the 23 January lockdown event at a level of 800,000 occurrences. It was, during that national emergency, the most frequently used term in all our data. After it reached its peak level, the usage tops slowly decreased to lower levels of 700,000, 500,000, and 400,000, reaching the latter level in the middle of February. Thereafter its use continued but at much lower levels. Its frequent use was supported by all media in the order commercial, social, and state (Table 3).

The five remaining Chinese coronavirus-related pneumonia names with a rise-and fall pattern were attested between 31 December 2019 and 23 January 2020. These early names were taken up by different protagonists in different segments of the media, while new names too started to circulate as soon as more insight about the disease was obtained. The next in line with a rise-and-fall pattern is name No. 8 *Wǔhàn fèiyán*, “Wuhan pneumonia”, which appeared in our data as early as 31 December 2019. The name remained dormant in our data until 23 January, when its usage jumped to a level of 90,000 hits. After that day, the frequency of use started to fall, reaching another small peak at 4 February of 40,000 before continuing to fall further to much lower levels of frequency (Figure 2). No. 8 “Wuhan pneumonia” was shared by commercial and social media and not used in official media. This is one of the first indications that Chinese governance is more oriented on international standards than on the needs of their own population (Table 5).

A related term is name No. 12 *Yěwèi fèiyán*, “Wild animal pneumonia”, which became a popular name after the role of Wuhan’s Huanan seafood market in the spread of the virus became an issue. The name was attested on 1 January. It jumped to a daily frequency use of 18,000 hits at 23 January, the lockdown announcement. The name had a relatively short support span and fell back to much lower levels after a week and continued that downward trend thereafter. It, too, was only supported by social and commercial oriented media, another indication of the orientation of official governance on international standards rather than on the communicative needs of Chinese citizens. The order this time was reversed with a majority use of 56% for social media (Table 4).

A similar, short-time-span fate awaited disease name No. 11 *Yěwèi bìngdú fèiyán*, “Viral pneumonia caused by wild animals”, a term more explicit than its predecessor No. 12 *Yěweì fèiyán* by the addition of the disease source *bìngdú*, “virus”. The term, as mentioned, got this more explicit name during its introduction in a CCTV program which created a short-term audience effect in the sense that other sources reported on this TV event. It only found a following in commerce-oriented sites and was not reported by social media (Table 5). The term was attested for the first time at 22 January, reached a maximum level of 3000 hits after which it fell back to a much lower level of 500 occurrences, and thereafter basically disappeared. Despite its official support, the No. 11 *Yěwèi bìngdú fèiyán* pneumonia name, in terms of frequency, merely had a marginal existence.

The next term of the rise-and-fall pattern is disease name No. 3 *Xīnxíng bìngdú fèiyán*, “Novel virus pneumonia”, which was attested as early as 9 January and was shared by all three media environments, while commerce-oriented media dominated its use. The name was only marginally supported by official and social media for a maximum of close to one-third of all occurrences for both state and social media combined (Table 3). The term hit a maximum of 6000 occurrences during the 23 January lockdown event, but after one week had fallen back to 2000 and slowly moved to lower levels until the end of the observation period in the middle of March, by which time it had basically disappeared in the data.

The last of the six Chinese rise-and-fall disease names is No. 10 *Hànfèi*, “Han P(neumonia)”, which appeared in social media on 21 January and reached its maximum of six occurrences during the 23 January lockdown event. It was neglected by official and commercial sites (Table 5). The name was invented by netizens looking for a short term that could cover all occurrences; a marginal but interesting example of name-giving developed by netizens in interaction with other site participants. The latter obviously did not like the term and basically ignored it (Figure 2).

The seventh and last term of the rise-and-fall type in our data is the English disease name No. 16 “NCP”, the English language abbreviation for “Novel Coronavirus Pneumonia”. The use of NCP was predominantly intended for a Chinese-speaking North American audience and appeared in commercial and social environments only, such as the popular Chinese channel “Los Angeles Life Interaction”. It started at a relatively high level of 25,000 occurrences, rapidly moving up to 30,000 hits, but thereafter slowly sank back to lower levels, reaching 5000 hits on 16 February, after which it lingered on for a while before sinking further to much lower levels.

An example of NCP will help to clarify its usage. We give an example from 22 March 2020 which was taken from Tencent’s WeChat social media account of the Jingxi Public Security department:

(5) NCP yìqíng fāshēng yǐlái, yǐ Xí Jìnpíng tóngzhì wéi héxīn de dǎng zhōngyāng gāodù zhòngshì.

“Since the outbreak of the *NCP*, the Party Central Committee with Comrade Xi Jinping at the core has attached great importance to it.”

(*Wēixìn gōngzhònghào Jìngxī Gōng’ān,* ‘WeChat public account *Jingxi* Public Security’ 22 March 2020).

All timelines discussed so far are summarized in Figure 2, where they are listed in their order of appearance.

A pattern with two peaks before gradual decline was observed for two of the coronavirus disease names. In order of appearance in our data, these were No. 13 *Bùmíng yuányīn bìngdúxìng fèiyán*, “Unknown viral pneumonia”, and No. 2 *Xīnxíng guānzhuàng bìngdú gǎnrǎn de fèiyán*, “Pneumonia caused by novel coronavirus”. The latter appeared on 10 January but reached a frequency peak at 23 January 2020, the date of the Wuhan lockdown, when its use jumped to a level of 140,000 thousand. It reached a second peak of 120,000 at 5 February, the announcement of 10,000 infections in Wuhan. Thereafter the frequency started to decline. For the two weeks of its popularity, it had an average daily use of 135,000 hits. The term was shared across all three media types. Nevertheless, like most names, it was most frequently used in commercial media. One in five of the remaining occurrences fell into each of the official and social domains.

We illustrate pneumonia name No. 2 with an example from the end of January 2020 taken from the China Youth Net site:

(6) Xīnxíng guānzhuàng bìngdú gǎnrǎn de fèiyán yǒu nǎxiē zhèngzhuàng?

“What are the symptoms of Pneumonia caused by the novel coronavirus?”

(*Zhōngguó Qīngnián Wǎng*; China Youth Net, 20 January 2020).

Frequencies for name No. 13 “Unknown viral pneumonia”, which appeared as early as 1 January, were much lower, not higher than 2500 daily uses. Two peaks followed each other between 4 and 13 January, shadowed by a lower peak of 2000 occurrences on 23 January, during the Wuhan lockdown announcement. It was a name formally approved by the authorities and occurring mainly in official media with a small following of 15% in social media. Commercial might not have liked the idea of “unknown”.

A fourth timeline pattern in our data is the names with a frequency peak at the end of the timeline. This pattern is illustrated by three disease names:

No. 7 *Xīnguān*, “Novel Coronavirus”;

No. 6 *Xīndú fèiyán*, “New viral pneumonia”; and

No. 14 *Zhōngguó fèiyán*, “China pneumonia”.

We will discuss them in this order. Name No. 7 “Novel Coronavirus” came into use around 20 January 2020 but reached its peak much later around 14 March, at a time when authorities started to relax movement in the province, another emotional event opening new ways of work, leisure, and entertainment. The occurrence peak rose sharply to over 100,000 tokens around that mid-March time and then fell back to a lower level of 60,000 with an average of 26,000 hits for the six weeks of its existence in our data. It was a popular term especially in commercial media, two-thirds of the occurrences were in that domain. The remaining one-third occurred in social media and we have no attested instances of use in the governmental domain for this more casual term.

Name No. 6 “New viral pneumonia” had some incidental occurrences since 29 January but showed a sudden peak of 150 occurrences in the week of February 20, which happened to be the date of the announcement of the lockdown extension until 10 March for all nonessential companies, including factories and schools. It is a term designed and fancied by state-controlled institutions, but had little support in commercial media and was totally neglected by social media (Table 5).

Name No. 14 *Zhōngguó fèiyán,* “China pneumonia”, appeared at the end of December 2019 for the first time but became more popular during the 23 January lockdown upheaval when it reached a daily frequency of more than 500, moving in the following weeks between 200 and 600 daily hits. In March 2020, that term experienced a sudden peak. This surge was the result of a press conference on 19 March 2020 (http://ling.cass.cn/xzfc/xzfc_xzgd/202002/t20200204_5084687.html)(accessed on 22 March 2021) by the then US President Donald Trump, who started to refer to the pandemic as the “Chinese virus”, a move that attracted media attention in various countries, including Chinese domestic media such as Tencent, («为什么新冠肺炎不能称为“中国肺炎”?» 3月22日 https://new.qq.com/rain/a/20200321A0CCYS00, accessed on 22 March 2021) Sohu, («“中国肺炎”之称用心险恶, 绝不容忍借疫情妖魔化中国!» 3月21日 https://www.sohu.com/a/381834410_730080, accessed on 22 March 2021) as well as China Finance and Economics («港科大学生会竟说出“中国肺炎”“东亚病flu”, 校长怒斥» 3月21日 http://www.cfi.net.cn/p20200321000027.html, accessed on 22 March 2021)These sources all reported on and criticized the president’s word choice. Trump’s controversial remarks led to heated discussions of “China pneumonia”, resulting in a sudden active period of this name variety.

Example (7) is an illustration of a message, in mid-March, spreading via Taiwanese media in which the erstwhile President Trump was criticized as being discriminatory as well as incompetent:

(7) Měiguó zǒngtǒng Tèlǎngpǔ chúle gěi bìngdú guànyǐ qíshìxìng de chēngwèi “zhōngguó fèiyán”, yǐjí xiànzhì Zhōng-Ou lǚkè fù Měi zhīwài, yìngduì yìqíng zhǐhuī wúfāng, yǔ fángyì zǒng zhǐhuī fù zǒngtǒng Péngsī hé jíkòng zhōngxīn zhījiān duōtóu mǎchē bù tóngdiào.

“In addition to labelling the virus ‘Chinese pneumonia’, which is discriminatory, and restricting travel to the United States by Chinese and European travelers, US President Trump has no means to respond to the epidemic. Vice-President Pence and the Center for Disease Control are not in tune!”

(*Zhōngguó Táiwān wǎng,* 20 March 2020, *Táihǎi wǎng tóngrì zhuǎnfā*; China Taiwan Net, 20 March 2020, forwarded on the same day by Taiwan Net)

The unwelcome nature of the name “China pneumonia” remained an issue also under medically informed netizens. An example from the end of March 2020 further shows the confusion that still existed, when a medical professional wondered what was wrong with a term like “Chinese pneumonia”:

(8) Wèishéme xīnguān fèiyán bùnéng chēngwéi “Zhōngguó fèiyán”?

“Why can’t the new Coronavirus pneumonia be called ‘China pneumonia’”?

(*Dīng Xiāng yīshēng;* Doctor Ding Xiang, 22 March 2020)

The last two Corona disease names are No. 9 *Wǔfèi*, “Wu(han) P(neumonia)”, and No. 1 *Xīnxíng guānzhuàng bìngdú fèiyán*, “Novel coronavirus pneumonia”. They have a similar timeline pattern, one quite different from the four patterns discussed so far. These patterns started low, slowly built up their frequency, reaching a peak in the middle of the observation period and then declined. Their frequencies, however, were of different magnitudes, name No. 1 reached frequencies close to 500,000, whereas name No. 9 had peaks limited to a few hundred hits. Given these frequency differences, we will start with name No. 1.

Disease name No. 1 *Xīnxíng guānzhuàng bìngdú fèiyán*, “Novel coronavirus pneumonia”, appeared on 9 January and remained unnoticed until the lockdown announcement on 23 January when it jumped up to 200,000 occurrences, slowly becoming more popular thereafter until it reached another high during the announcement of lockdown extensions on 13 and 14 February, reaching a high of 500,000 daily occurrences. After that peak, its use started to decline, slowly falling back to a very low level at the end of the data collection period. Its popularity was supported by all media types with dominance of commerce, followed by governmental and social media, in that order (Table 3).

Name No. 9, *Wǔfèi*’s daily frequency was relatively low, varying between 100 and 300 with usage equally shared between commercial and social media (Table 5). Attested as early as 1 January, the name reached its first frequency peak of close to 200 hits during the lockdown announcement on 23 January and thereafter reached a succession of higher peaks at mildly higher levels of 300 and 400, the latter occurring during the lockdown extensions of 13 and 20 February referred to already.

Timelines were presented in order of appearance, see Figure 2 and Table 2 for overviews. Peak events were related to official announcements, the Wuhan lockdown, and related events. We presented five timeline patterns, rise and high frequency, rise-and-fall, double initial peak, end peak, and middle peak. The rise-and-fall timelines showed a peak followed by a fall. Three of these showed a slow fall, whereas the remaining three were examples of rapid rises and rapid falls, leaving a special position for NCP with a not too rapid but still clear fall. The two double peaks also occurred at the beginning of the timeline. If these double peak timelines are seen as a special case of rise-and-fall, this latter pattern is dominant with 56% of all data. The three end peaks each had their own story, with No. 11 “Viral pneumonia caused by wild animals” showing the effect of media attention and No. 14 “China pneumonia” demonstrating the impact of international influences on the frequency of use of Chinese pneumonia names.

### 4.3. Frequency and Term Length

The 16 names in our sample have a different number of syllables or in China speech, characters, which raises the question as to why, if shorter names are available, people would choose long and cumbersome descriptive names? In Chinese this is a relevant question since in the language a preference exists for four-character combinations of which there are collections, called *chengyu*, “idioms”, containing thousands of examples. An illustration is the proverb *Jiùde bú qù, xīnde bù lái*, “If the old does not go, the new won’t come”, which as the reader can see contains two four-character expressions. Among the 14 Chinese pneumonia names studied, four-character names, too, were most popular, represented by six names or 42% of the data. Three names were abbreviated in the form of two-characters, leaving five or one-third of the names in a longer form of six characters or more (Table 6).

The question raised now is the extent to which longer names in comparison to four-character names were used at lower frequencies, a result which would show a preference for the latter. To check this possibility, a correlation coefficient was calculated for the four most frequent multicharacter names and the first four most frequent four-character names. This calculation showed a strong correlation between the two name-types (*p* < 0.003, one-tail). This result showed that the hypothesis that four-character names will acquire higher frequencies than longer names needs to be rejected. Names longer than four-characters had a frequency pattern which was strongly similar to that of the four-character names. Variation in the calculation by choosing somewhat different, less frequent four-character names, did not change that picture, a remarkable result from the perspective of communication theory, which in this case would expect a preference for four-character expressions, supported in that by grammatical patterns existing in the language. The correlation data of the massive figures collected for this study showed that the hypothesis needs to be rejected. (The probability was calculated with the help of the statistics calculator for correlations at danielsoper.com).

**Figure 3:**
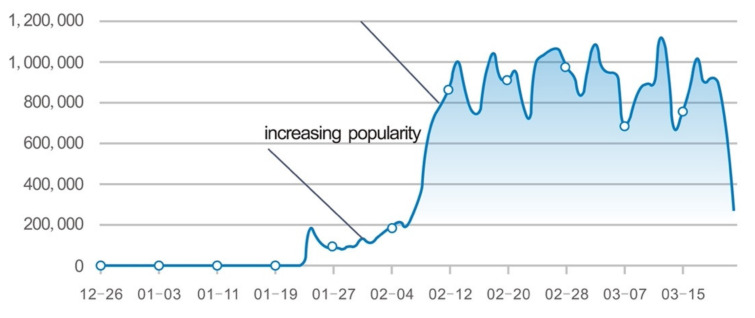
Increase in popularity of name No. 5 *Xīnguān fèiyán* between the end of January and middle of February 2020.

Name No. 2 *Xīnxíng guānzhuàng bìngdú fèiyán* had a double peak rise-and-fall pattern. It reached a first peak of 140,000 hits on 23 January and a second peak of 120,000 on 5 February, the announcement of 10,000 infections in Wuhan. At the time name No. 2 *Xīnxíng guānzhuàng bìngdú fèiyán* reached its first peak, name No. 5 *Xīnguān fèiyán* had already become more popular with 200,000 hits. The name is an abbreviation of name No. 2, as will be clear; it contains the two main components of the disease, *xīnguān*, “the new corona(virus)”, and *fèiyán*, “pneumonia”, but also had the preferred Chinese word format of a four-character expression. The name occurred millions of times in our data. The timeline shows a peak at 23 January and after that date it grew in popularity until it reached the one million hits level. We consider this slow but incremental growth in popularity as proof of the importance of the preferred four-character word form for a Chinese disease name. For clarification we took the graph of the timeline from Figure 2 and reproduced it here with focus on the growth pattern (Figure 3).

## 5. Discussion

Our data show that naming a disease needs to take into consideration the timeline along which the various names were introduced, the media type involved, the shape of the timeline of each individual name, as well as the type of word format applied. Our frequency data, in addition, provide the necessary information for an empirical evaluation of the various names used. In the following, we perform such an evaluation using all five criteria and show that the variation in our data was not random: it was steered by official media, and by the National Health Commission, but also developed pragmatically over time in commercial and social media.

By 23 January, during the Wuhan lockdown, five terms attracted the most attention, four official terms and one “practical” term, “Wuhan pneumonia”. Competition among the four official names finally led to the dominance of name No. 5 *Xīnguān fèiyán*, “New coronavirus pneumonia”. The path to that dominance will be the first issue in our discussion, the result of which we will relate to the hypotheses we formulated, event dependence of keyword frequency, confusion by name varieties, and global linguistic governance.

One question that readers might have in this context is the potential influence of censorship on our data. Clearly, as concluded by Zhu et al. [15] and Liu et al. [16], the media gave insufficient attention to the new disease in the beginning weeks of January and this can be directly related to communication rules prescribed in China by communist party bodies. Communication along prescribed lines is encouraged, whereas information spreading in chatgroups that is considered harmful is suppressed. Our data, too, confirm the relatively limited attention to the disease at the beginning of January as reported by the two earlier studies [15,16], and, apart from the lack of journalistic attention, this could have been the effect of (self-)censorship among netizens. However, the disease terms we selected have no relation with any harmful or forbidden content and, therefore, it is extremely unlikely that censors might have noticed anything strange or prohibitable in the various disease names we selected. During the emerging epidemic, there were no more urgent concerns to address than the disease spreading and people dying.

Some studies [6,10] contained the idea that a variety of disease names create confusion for laypeople. The names in our data set that were used for the new corona disease do not support this view. All name choices at the end of December 2019 and in January 2020, the time China’s National Health Commission instructed the Wuhan Municipal Health Commission to announce the outbreak, were related to the form of “pneumonia” concerned with names such as “new type”, “Chinese”, “Wuhan”, “unknown”, and “game (wild animals)”. Each of these choices is practical and informative for netizens countrywide. Online readers learned that this form of pneumonia that was discovered in “Wuhan” was “new”, limited to “China” by that time, had an “unknown” origin, and had some relation with the Wuhan “seafood market”.

Of the six early terms, by the end of December 2019 and the following 1 January, two were used in official media, name No. 4 *Xīnxíng fèiyán*, “New type pneumonia”, which noted that there was now a “new type” of pneumonia in Wuhan, and name No. 13 *Bùmíng yuányīn bìngdúxìng fèiyán*, “Unknown viral pneumonia”, which recognized that this “new type” was not yet identified. Commercial and social media used four more terms in those early days:

No. 8 *Wǔhàn fèiyán*, “Wuhan pneumonia”;

No. 12 *Yěwèi fèiyán*, “Wild animal pneumonia”; and

No.9 *Wǔfèi*, “Wu P(neumonia)”.

Name No. 14 *Zhōngguó fèiyán*, “China pneumonia”, which appeared in our data on 31 December, was used by two Taiwanese websites making clear that this was a mainland Chinese and not a Taiwanese problem. Taiwan’s reaction to the new viral disease has stood out since, mainly for political reasons.

Of these four terms, three have the preferred word form of a four-character expression, whereas No. 9 *Wǔfèi* is an example of an abbreviation to a two-character expression. The case of these four names makes clear that in China administrative bodies stick with WHO guidelines for disease names by avoiding names of cities or locations, whereas commercial and social media used these terms with great enthusiasm in this period of distress. The WHO acted against regional naming, considering it a form of regional stigmatization, which makes perfect sense. It advocated “depoliticization” in order to help to eliminate the negative impact of disease naming. The 2015 WHO file (https://www.who.int/mediacentre/news/notes/2015/naming-new-diseases/zh/, accessed on 22 March 2021) *Best Practices for Naming New Human Infectious Diseases* states that “When naming, avoid geographic information such as cities, countries, regions, and continents”.

The relation between global governance and official names selected for the “new type pneumonia” shows that WHO guidelines were adhered to by Chinese administrative bodies but did not restrict disease naming in commercial media and social networks. As we have seen already, of the six names attested at or before 1 January 2020, two were official names and the remaining four “practical” names, which were only used in commercial and social media. After 1 January, eight further names for the pathogen appeared. Six of these eight were names used in official media, reflecting changes in understanding of the pathogen and its infectious character. The National Health Commission on 7 February 2020 declared names No. 2, No. 1, and No. 5 official names. All three were high frequency names, as we have seen (Figure 2; Table 6). For the sake of clarity, we reproduce them here for ease of reference:

No. 2 *Xīnxíng guānzhuàng bìngdú gǎnrǎn de fèiyán*, “Pneumonia caused by novel coronavirus”;

No. 1 *Xīnxíng guānzhuàng bìngdú fèiyán*, “Novel coronavirus pneumonia”; and

No. 5 *Xīnguān fèiyán*, “New coronavirus pneumonia”.

On that date, too, NCP was declared an official English name as an abbreviation of ‘Novel Coronavirus Pneumonia’. This name, however, was deleted after the official WHO name of COVID-19 was announced on 11 February. The point we want to make is that after 8 February, the date on which the announcement appeared on the website of the National Health Commission, official media strictly followed this ruling, confirming the integration of global and local governance of the disease names.

Before 7 February, however, on 23 January to be precise, the Wuhan lockdown was announced, during which 10 of the 14 Chinese terms for the disease obtained relatively high daily frequencies (Table 6; Figure 2). Five of these had very high frequencies, the remaining five did flare up but at much lower and often marginal frequencies. The five names with very high daily frequencies during the lockdown were the three names mentioned above as official names together with the earliest official name, name No. 4 *Xīnxíng fèiyán*, “New type pneumonia”, as well as another early name, name No. 8 *Wǔhàn fèiyán.* The latter name, which should not be used according to WHO insight, had a daily frequency of 80,000 on that day, not as much by some margin as name No. 4 with 800,000 hits, or the three official names No. 2, No 1, and No. 5 with scores of 200,000 and 120,000 daily uses, but still a considerable number. During the lockdown of the city of Wuhan, this name seemed to be a clearer and more practical indication.

Names that were not popular during the lockdown were name No. 14 “China pneumonia”, as observed, a name mainly used outside China, and abbreviations such No. 10 *Hànfèi*, No. 9 *Wǔfèi*, and No. 7 *Xīnguān*, the latter an abbreviation of name No. 5 that only obtained higher daily frequencies of up to 100,000 by the middle of March. By 23 January, the focus was on Wuhan and not yet on China and abbreviations as a form of casual writing did not seem to be appropriate ways of expressing oneself and that applied to commercial media and, to a lesser extent, to social media as well.

Our empirical data allow a distinction between state governance, governance by commercial organizations, and network governance. The Chinese state in the past 40 years had developed a system of governance that in theory was capable of handling a new viral disease. It had learned its lesson from the 2003 SARS (severe acute respiratory syndrome) epidemic when worldwide 774 people reportedly died of the disease (Revised U.S. Surveillance Case Definition for Severe Acute Respiratory Syndrome (SARS) and Update on SARS Cases --- United States and Worldwide, December 2003. MMWR weekly, 12 December 2003, 52(49), 1202–1206). Governance bodies relevant during the “new coronavirus” outbreak were the Wuhan Health Commission, Hubei province’s Centre for Disease Control and Prevention (CDC) with at the national level the China National Health Commission and the National CDC, an organizational structure that should be able to control a new viral disease outbreak.

In contrast with state governance, commercial governance is strategically directed at corporate goals for which the management is held accountable. It is therefore no surprise that we find a direct relationship between the terminological choices of commercial media and the terms found in social networks. State policies and administrative procedures influence both, but in everyday affairs commercial and social media tend to express themselves in similar fashion, since companies and customers tend to process information in very practical ways. This practicality of commercial and social media is one of our core findings, which leads to terminological choices that governmental bodies and state media find inappropriate.

The data we presented from the perspective of “language governance” suggest that a system should be designed for alerting the development of serious medical conditions when confronted with an outbreak of a sudden “unknown” disease. The language governance issue can only become relevant after hospital care, virus identification, genetic sequencing, and infectiousness have been addressed. Even though this information was available at the end of January 2020, global governance failed in our view since the announcement of a pandemic occurred only on 12 March 2020, a date that can easily be argued as being far too late (https://www.euro.who.int/en/health-topics/health-emergencies/coronavirus-covid-19/news/news/2020/3/who-announces-covid-19-outbreak-a-pandemic, accessed on 20 July 2021). An analysis of this aspect of global governance obviously is outside the scope of this paper and needs to be addressed elsewhere.

Our media data confirmed the influence of three main societal forces, the Chinese state, commerce, and netizen communities. Commercial enterprises with their financial power and media presence were the largest consumer of coronavirus names. The Chinese state made certain names official, thereby creating a following in official circles, but had limited influence as regards the number of terms introduced and supported in commercial and social media. Netizen communities did not form a single coherent force but supported certain terms in favor of their main position as consumers and commentators. Civil society has proven to be sufficiently flexible as regards name-giving, before their choices are scrutinized by national or global sources.

Studies that promoted the idea of name confusion can use the empirical data presented in this paper to reassess their own findings. The rather strict and explicit considerations by the WHO also should take contemporary research into consideration. For those who consider the number of names selected to be too small, the option is open to repeat the study with terms they consider better suited. Students of governance, too, can use our empirical data to see to what extent their proposals are coherent and applicable. We generally hope that our study helps to clarify the forces at work in disease name selection during an emergency outbreak. Our study took place during such an emergency outbreak and we worked during a lockdown. Further research can address less chaotic circumstances over a longer period of time.

## 6. Conclusions

The data presented in this study were collected online with the help of the Qingbo Big Data site, a popular big data platform for Chinese news media. In all, we selected 16 disease names and collected more than 88 million sentence tokens, together forming the basis of our data. When we used data to test the first “peak and wane” hypothesis, the timeline data confirmed the driving force of main news events as reported in earlier studies [15,16]. Disease name keywords, however, are not cumulative but in competition with each other and factors such as content clarity, word form type, and frequency shape the best candidate(s). The second hypothesis, the “confusion” hypothesis, has been rejected. The disease names in use reflected the needs of the media participants. Officially backed terms were mostly supported, but commercial and social media created their own practical names and shared most of the terms in their networks. The third hypothesis, the “linguistic incompetence” in global settings hypothesis, was also rejected. State media are very well capable of interacting at the global level with medical authorities and bodies. State media did not accept terms such as “Wuhan pneumonia”, being guided by a WHO publication on best practices for naming infectious diseases, which had a strong impact on the governance of administrative practices in China. We conclude that WHO guidelines are dogmatic and should be adjusted by supporting early warning procedures in local languages where practical informative names are crucial for all participants and stakeholders.

## Figures and Tables

**Table 1:** List of 16 COVID-19 names selected; coronavirus disease name survey 2020.

No.	Pinyin	English Name Used in This Paper	Original Language
1	*Xīnxíng guānzhuàng bìngdú feìyán*	Novel coronavirus pneumonia	Chinese
2	*Xīnxíng guānzhuàng bìngdú gǎnrǎn de feìyán*	Pneumonia caused by novel coronavirus	
3	*Xīnxíng bìngdú feìyán*	Novel viral pneumonia	
4	*Xīnxíng feìyán*	New type pneumonia	
5	*Xīnguān feìyán*	New coronavirus pneumonia	
6	*Xīndú feìyán*	New viral pneumonia	
7	*Xīnguān*	Novel coronavirus	
8	*W* *ǔh* *àn fe* *ìy* *án*	Wuhan pneumonia	
9	*W* *ǔfeì*	Wu P	
10	*Hànfeì*	Han P	
11	*Yěweì bìngdú feìyán*	Viral pneumonia caused by wild animals	
12	*Yěweì feìyán*	Wild animal pneumonia	
13	*Bùmíng yuányīn bìngdúxìng feìyán*	Unknown viral pneumonia	
14	*Zhōngguó feìyán*	China pneumonia	
15		COVID-19 (Corona virus disease 2019)	English
16		NCP (Novel coronavirus pneumonia)	

Note: The two English names, COVID-19 and NCP, were used in this form in certain Chinese communications. The 14 “English translations” were converted from the original Chinese by the current researchers.

**Table 2:** List of coronavirus disease names according to date of first occurrence; coronavirus disease name survey 2020.

Name No.	Date	English Name
No. 4 *Xīnxíng fèiyán*	30 December 2019	New type pneumonia
No. 14 *Zhōngguó fèiyán*	31 December 2019	China pneumonia
No. 8 *Wǔhàn fèiyán*		Wuhan pneumonia
No. 13 *Bùmíng yuányīn bìngdúxìng fèiyán*	1 January 2020	Unknown viral pneumonia
No. 12 *Yěwèi fèiyán*		Wild animal pneumonia
No. 9 *Wǔfèi*		Wu P(neumonia)
No. 1 *Xīnxíng guānzhuàng bìngdú fèiyán*	9 January 2020	Novel coronavirus pneumonia
No. 3 *Xīnxíng bìngdú fèiyán*		Novel viral pneumonia
No. 2 *Xīnxíng guānzhuàng bìngdú gǎnrǎn de fèiyán*	10 January 2020	Pneumonia caused by novel coronavirus
No. 5 *Xīnguān fèiyán*	18 January 2020	New coronavirus pneumonia
No. 6 *Xīndú fèiyán*	20 January 2020	New viral pneumonia
No. 7 *Xīnguān*		Novel coronavirus
No. 10 *Hànfèi*	21 January 2020	Han P(neumonia)
No. 11 *Yěwèi bìngdú fèiyán*	22 January 2020	Viral pneumonia caused by wild animals
No. 16	08 January 2020	COVID-19
No. 15	11 January 2020	NCP (novel coronavirus pneumonia)

**Table 3:** Frequency and percentage distribution of six terms shared across channels; coronavirus disease name survey 2020.

Name		State	Commercial	Social	Freq. (Thousand) *
No. 5	*Xīnguān fèiyán*	42	44	14	4500
No. 4	*Xīnxíng fèiyán*	12	50	38	1900
No. 1	*Xīnxíng guānzhuàng bìngdú fèiyán*	28	55	17	1800
No. 2	*Xīnxíng guānzhuàng bìngdú g* *ǎnr* *ǎn de fèiyán*	20	60	20	300
No. 3	*Xīnxíng bìngdú fèiyán*	15	70	15	10.4
No. 14	*Zhōngguó fèiyán*	28	55	17	3.3

* Total of weekly averages across eight weeks.

**Table 4:** Frequency and percentage distribution of six terms shared by commercial companies and social media; coronavirus disease name survey 2020.

Name	State	Commercial	Social	Freq. (Thousands) *
No. 7 *Xīnguān*	--	68	32	161
No. 8 *Wǔhàn feìyán*	--	58	42	124
No. 15 COVID-19	--	85	15	95
No. 16 NCP	--	85	15	60
No. 12 *Yěwèi fèiyán*	--	44	56	22
No. 9 *Wǔfèi*	--	50	50	1.2

* Total of weekly averages across eight weeks.

**Table 5:** Frequency and percentage distribution of the remaining four terms used by state, commercial, and social media.

Name	State	Commercial	Social	Frequency *
No. 13 *Bùmíng yuányīn bìngdúxìng fèiyán*	82	--	18	3200
No. 11 *Yěwèi bìngdú fèiyán*	60	40	--	3000
No. 6 *Xīndú fèiyán*	71		29	150
No. 10 *Hànfèi*	--	--	100	19

* Total of weekly averages across eight weeks.

**Table 6:** Frequency and percentage distribution of the 16 pneumonia names; coronavirus name study 2020.

Name	State	Commercial	Social	Freq. (1000) *
No. 5 *Xīnguān fèiyán*	42	44	14	4500
No. 4 *Xīnxíng fèiyán*	12	50	38	1900
No. 1 *Xīnxíng guānzhuàng bìngdú fèiyán*	28	55	17	1800
No. 2 *Xīnxíng guānzhuàng bìngdú gǎnrǎn de feìyán*	20	60	20	300
No. 7 *Xīnguān*	--	68	32	161
No. 8 *Wǔhàn fèiyán*	--	58	42	124
No. 15 COVID-19	--	85	15	95
No. 16 NCP	--	85	15	60
No. 12 *Yěweì fèiyán*	--	44	56	22
No. 3 *Xīnxíng bìngdú feìyán*	15	70	15	10.4
No.14 *Zhōngguó fèiyán*	28	55	17	3.3
No. 13 *Bùmíng yuányīn bìngdúxìng fèiyán*	82	--	18	3.2
No. 11 *Yěweì bìngdú fèiyán*	60	40	--	3
No. 9 *Wǔfèi*	--	50	50	1.2
No. 6 *Xīndú fèiyán*	71	29	--	0.15
No. 10 *Hànfèi*	--	--	100	0.019

* Total of weekly averages across eight weeks.

## Data Availability

Not applicable.
